# Population Muscle Strength Predicts Olympic Medal Tallies: Evidence from 20 Countries in the PURE Prospective Cohort Study

**DOI:** 10.1371/journal.pone.0169821

**Published:** 2017-01-20

**Authors:** Darryl P. Leong, Martin McKee, Salim Yusuf, on behalf of PURE Investigators

**Affiliations:** 1 The Population Health Research Institute, McMaster University and Hamilton Health Sciences, Hamilton, Ontario, Canada; 2 London School of Hygiene and Tropical Medicine, London, United Kingdom; St. Michael's Hospital, CANADA

## Abstract

**Background:**

National sporting achievement at the Olympic Games is important for national pride and prestige, and to promote participation in sport. Summer Olympic Games medal tallies have been associated with national wealth, and also social development and healthcare expenditure. It is uncertain however, how these socioeconomic factors translate into Olympic success. The objective of this study was therefore to examine the relationship between population muscle strength and Olympic medal tallies.

**Methods and Results:**

This study of handgrip strength represents a cross-sectional analysis of the Prospective Urban Rural Epidemiology (PURE) study, which is an ongoing population cohort study of individuals from high-, middle-, and low-income countries. Within participating countries, households from both urban and rural communities were invited to participate using a sampling strategy intended to yield a sample that was representative of the community. Households were eligible if at least one member was aged 35–70 years and if they intended living at the same address for a further four years. A total of 152,610 participants from these households, located in 21 countries, were included in this analysis. Handgrip strength was measured using a Jamar dynanometer. Olympic medal tallies were made over the five most recent Summer Games.

There was a significant positive association between national population grip strength (GS) and medal tally that persisted after adjustment for sex, age, height, average daily caloric intake and GDP (total and per capita). For every 1kg increase in population GS, the medal tally increased by 36% (95% CI 13–65%, p = 0.001) after adjustment. Among countries that won at least one medal over the four most recent Summer Olympic Games, there was a close linear relationship between adjusted GS and the natural logarithm of the per capita medal tally (adjusted r = 0.74, p = 0.002).

**Conclusions:**

Population muscle strength may be an important determinant of Summer Olympic Games medal success. Further research is needed to understand whether population muscle strength is modifiable, and whether this can improve Olympic medal success. Extreme outcomes may reflect the average attributes of the population from which the individual experiencing the extreme outcome is drawn.

## Introduction

The Olympic Games are the pinnacle of sporting contests for many athletes, and capture the attention of the world every four years. The Olympic Creed reads, “The most important thing in the Olympic Games is not to win but to take part…..The essential thing is not to have conquered but to have fought well.” Notwithstanding this noble sentiment, Olympic medals are, to a large extent, the standard by which the success of athletes and of their countries has been judged. Medal tallies are almost invariably the final statistic presented in any Olympic Games news report, and medallists are transformed overnight into fêted celebrities.

Considerable research and resources are devoted to identifying the factors that give athletes the advantage needed to become an Olympic medallist. A nation’s medal success is likely to reflect its pool of athletic talent and ability to identify and nurture gifted athletes with sophisticated training techniques. Thus, more populous and wealthy countries do well[1], although the effects vary between summer and winter games, with national income more important for the latter along with, unsurprisingly, climate and geography (with countries that have Alpine conditions doing well at skiing and related sports) [2]. Sending more athletes helps, as does spending more on health[3] and social development[4]. More controversially, it has been suggested that culture and religion may play a role[5]. Politics also matters, with research covering the games since 1952 finding that single party and communist regimes did particularly well[2]; the authors of that paper noted that those countries may have a “different approach to participation, training and incentives for success”. There are many contemporary exceptions to these factors that are associated with Olympic medal success. India, despite being the most populous country in the world, has done relatively poorly in terms of winning Olympic medals[6], the “Asian dragons” underperform relative to their populations and economies while the USA and China do better than expected[7], and some quite small countries (e.g. Jamaica) do very well[8].

One may consider Olympic athletes to lie on an extreme of a Gaussian curve for any relevant physical characteristic, be it strength, speed, or endurance. The values at the extremes reflect the mean values in a population, and so this led us to explore whether there are differences in mean levels of physical characteristics in the population among countries and whether these mean levels could predict their ranking in terms of Olympic medals.

We examined this hypothesis using data from the Prospective Urban Rural Epidemiology (PURE) study. The PURE study is an ongoing prospective cohort study of more than 154,000 community-based individuals from 21 low-, middle- and high-income countries and states.

## Methods

### Study Design and Participants

The design of the PURE study has been described previously[9]. In brief, participating countries were selected to represent significant socioeconomic heterogeneity. For reasons of feasibility, proportionate sampling of all countries worldwide, or of regions within countries, was not undertaken. Countries selected were Canada, Sweden, United Arab Emirates, Saudi Arabia, Argentina, Brazil, Chile, China, Colombia, Iran, Malaysia, Poland, South Africa, Turkey, Philippines, Palestine, Bangladesh, India, Pakistan, and Zimbabwe. In each country, both urban and rural communities were selected because of anticipated variation in socioeconomic and physical environments between the two settings.

The sampling approach, also described in detail previously [9], involves subjects being selected in a three stage sampling process, selecting “communities”, then households within them, and finally individuals within households. The community is defined as a group of people who have common characteristics and reside in a defined geographic area. Our sampling goal was to enrol an unbiased sample of households within each community, although the precise method varied, consistent with local practice in undertaking surveys, which takes account of local infrastructure (such as postal services) and customs (such as the use of community leaders as intermediaries). At least three attempts were made to reach each household. Households were eligible if at least one member was aged 35–70 years and household members intended to stay at that address for a further four years.

This sampling approach has yielded estimates of mortality rates that correlate closely with United Nations’ estimates [10]. The PURE study and its consent procedures and form was approved by the relevant research ethics committees in participating countries (please refer to S2 Appendix for the full list). This study was conducted in accordance with the principles of the Declaration of Helsinki. Written informed consent was obtained from all participants prior to their participation. A signed copy of the consent form is kept in each participant’s file at the site and a copy is provided to the participant.

### Subject Characteristics

Participants completed a standardised evaluation, including measurement of anthropometrics. Muscle strength was evaluated by grip strength (GS), which was measured using a Jamar dynanometer according to a standardised protocol. For the first study participants, three measurements were made from the participant’s non-dominant hand. During the course of the study, the protocol was amended so that three measurements were made from both hands of each participant. For the present analysis, we used only the maximum values obtained from each hand[11]. Where values were missing for one hand but present for the other, we imputed values for the missing hand using the coefficient and constant from the linear regression of non-dominant and dominant hand GS. In total, 39,765 dominant hand GS values, and 1207 non-dominant hand GS values were imputed. Overall individual GS was then calculated as the mean of non-dominant and dominant hand GS. Both the unadjusted mean GS, and the GS adjusted for age, sex, height, and daily dietary caloric intake were subsequently calculated stratified by country.

### Medal Counts and Other Country Characteristics

Summer Olympic Games medal tallies were made by summing all medals won by each country during the most recent five Olympic Games (Rio de Janeiro 2016, London 2012, Beijing 2008, Athens 2004, and Sydney 2000)[12]. National population estimates[13] and gross domestic product (GDP)[14] were recorded as of 2012.

### Statistical Analysis

Categorical variables are presented as count (percentage), and continuous variables as mean±standard deviation (SD), or median (25^th^-75^th^ percentile). The relationship between medal tally (expressed per million citizens), and both mean unadjusted GS, and GS adjusted for age, sex, height, and daily caloric intake was evaluated by Poisson regression. Further adjustment was then made for country/state GDP. In a separate sensitivity analysis, the relationship between medal tally and GS was adjusted for age, sex, height, daily caloric intake, and country/state *per capita* GDP. Model fit was evaluated using the Pearson’s goodness-of-fit statistic. For countries that won at least one medal, the relationship between the natural logarithm of the medal tally (per million citizens), and both mean unadjusted GS, and GS adjusted for age, sex, height, and daily caloric intake was also evaluated by linear regression. In further sensitivity analyses, these regressions were repeated with gold medal tally as the outcome variable.

In separate analyses, using Poisson and linear regression, we also evaluated whether a) height, b) body mass index (BMI), or c) country GDP was associated with the natural logarithm of the medal tally. Statistical analysis was performed using STATA 13.1 (StataCorp, College Station, TX, US). A two-tailed p-value of <0.05 was considered statistically significant for all analyses.

## Results

At the time of writing, 180,947 individuals were enrolled in the PURE study, among whom GS was recorded for 152,610 (84%). Subject characteristics stratified by country, together with country populations and GDP, are presented in Table 1. In the 2000, 2004, 2008, 2012, and 2016 Olympic games, the PURE countries collectively won respectively 14%, 15%, 19%, 20%, and 19% of the medals awarded.

**Table 1:** PURE subject characteristics stratified by country/state. BMI = body-mass index; GDP = gross domestic product; GS = grip strength; NA = not available because dietary data have not yet been analysed; SD = standard deviation. Medal tally refers to Summer Olympic Games from 2000–16 inclusive.

Country/ State	Population in 2012	GDP in 2012 (millions of USD)	Medal tally	Male sex, n (%)	Median age (25^th^-75^th^ percentile), years	Mean height± SD, cm	Mean BMI±SD, kg/m2	Mean GS±SD, kg	Adjusted* GS (95% CI), kg
Argentina N = 7462	42,192,500	603,153	24	2875 (39)	51 (43–59)	164±9.5	29.6±6.22	33.0±11.3	32.8 (32.6–32.9)
BangladeshN = 2712	161,083,808	116,034	0	1232 (45)	45 (38–52)	156±8.4	21.9±4.20	26.2±9.62	26.7 (26.4–26.9)
Brazil N = 5575	199,321,400	2,248,781	73	2493 (45)	52 (45–59)	162±9.3	27.9±5.20	35.4±10.0	35.0 (34.8–35.2)
Canada N = 10,010	34,300,080	1,821,446	84	4655 (47)	54 (46–61)	168±9.5	27.7±5.76	35.9±12.4	34.2 (34.1–34.4)
Chile N = 3218	17,067,370	266,259	5	1114 (35)	52 (44–60)	158±8.8	29.9±5.34	25.9±10.9	28.1 (27.8–28.3)
China N = 46,890	1,343,240,000	8,229,491	379	19,528 (42)	51 (43–58)	161±8.2	24.6±4.03	31.8±10.3	32.1 (32.1–32.2)
Colombia N = 7475	45,239,080	370,328	21	2685 (36)	50 (43–58)	159±9.0	26.4±5.01	29.4±10.9	30.8 (30.6–30.9)
India N = 25,786	1,205,074,000	1,858,745	13	11,279 (44)	47 (40–56)	158±9.1	23.0±5.04	26.6±8.66	26.7 (26.6–26.8)
Iran N = 6011	78,868,710	502,729	32	2876 (48)	47 (41–55)	162±9.7	27.3±4.67	33.0±11.4	31.6 (31.4–31.8)
Malaysia N = 10,542	29,179,950	305,033	8	4513 (43)	51 (44–59)	157±8.6	26.6±5.18	26.1±9.88	27.1 (26.9–27.3)
Pakistan N = 1824	190,291,100	224,880	0	896 (49)	45 (40–53)	161±9.1	24.0±5.82	20.0±10.1	18.2 (17.8–18.6)
Palestine N = 1561	4,047,000	11,279	0	781 (50)	48 (41–56)	164±9.8	30.3±6.32	33.4±10.7	31.5 (31.1–31.8)
Philippines N = 4659	103,775,000	250,182	1	1346 (29)	53 (45–60)	156±7.9	24.2±4.61	25.1±8.26	NA
Poland N = 2020	38,415,280	490,213	55	754 (37)	55 (48–61)	165±8.9	28.1±5.08	35.8±12.4	36.0 (35.7–36.3)
Saudi Arabia N = 2046	26,534,500	733.956	3	1165 (57)	45 (39–52)	164±8.9	30.7±5.98	30.7±10.9	27.6 (27.2–27.8)
South Africa N = 3238	48,810,430	382,338	28	1030 (32)	49 (42–57)	160±8.2	27.0±8.41	24.5±12.6	25.9 (25.6–26.1)
Sweden N = 4097	9,103,788	523,941	43	1932 (47)	53 (46–60)	172±9.3	26.5±4.18	39.0±12.2	36.0 (35.8–36.3)
Turkey N = 4058	79,749,460	788,863	37	1600 (39)	49 (43–57)	161±9.2	30.5±6.02	30.6±10.6	30.8 (30.6–31.0)
UAE N = 914	5,314,317	383,799	2	262 (29)	48 (40–57)	160±8.8	30.2±6.40	27.6±9.23	28.7 (28.2–29.2)
Zimbabwe N = 815	12,619,600	12,472	7	230 (28)	52 (45–60)	162±7.8	24.6±6.09	30.1±8.19	31.5 (31.0–32.0)

* Adjusted for age, sex, height, and daily caloric intake.

The Poisson regression for the association between GS and medal tally is presented in Table 2. This demonstrates a consistent positive association between national GS and medal tally. As a sensitivity analysis, we repeated the Poisson regression model adjusting for age, sex, height, daily caloric intake, and per capita national GDP. This analysis did not meaningfully affect our findings: the percent increase in expected medal count was 28% (95% CI 6–56%, p = 0.011) for each 1kg increase in national GS. Model fit was appropriate (the Pearson goodness-of-fit statistic was 6.4, p = 0.98, for the model including height, age, sex and national GDP). When the Poisson regression was repeated on the gold medal tally, the coefficient point estimates were similar, although they were not statistically significantly different from the null owing to wider confidence intervals. The respective percent increases in expected gold medal counts per 1kg increase in GS were respectively 29% (p = 0.08), 28% (p = 0.07), and 35% (p = 0.1) for models unadjusted, adjusted for national GDP, and adjusted for age, sex, height, daily caloric intake and national GDP.

**Table 2:** Poisson regression models for the association between grip strength, and national Olympic per capita medal tally. Abbreviations as per Table 1.

Model	Percent increase in expected medal count (95% confidence interval) per 1kg increase in GS	p-value
Unadjusted	31 (13–51)	<0.001
Adjusted for national GDP	29 (13–48)	<0.001
Adjusted for age, sex, height, and daily caloric intake	38 (13–69)	0.002
Adjusted for age, sex, height, daily caloric intake and national GDP	36 (13–65)	0.001

**Fig 1:**
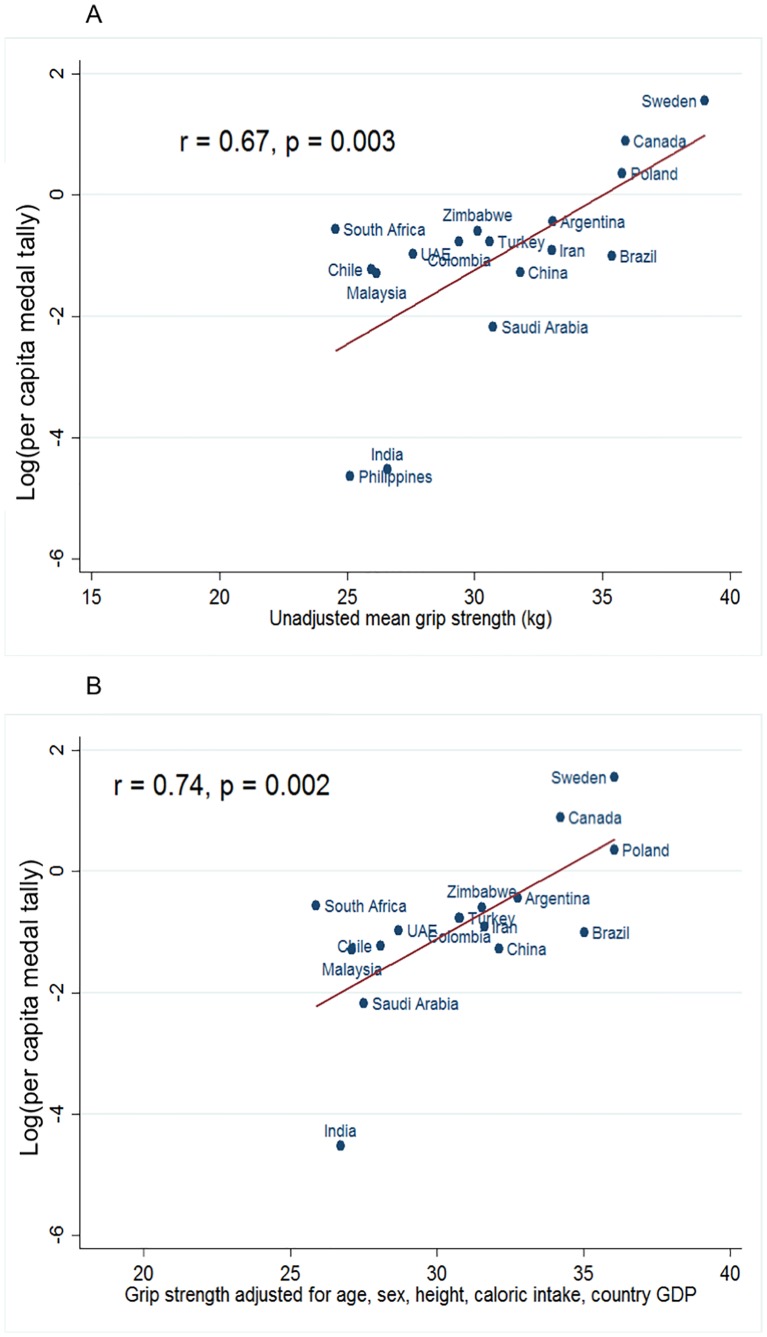
Scatter plots depicting the relationship between mean national grip strength. The relationship between mean national grip strength and national GDP, and the natural logarithm of the per capita medal tally: (A)–unadjusted, and (B)–adjusted for age, sex, height, dietary caloric intake and national GDP), and the natural logarithm of the per capita medal tally. Countries that did not win any summer Olympic medals from 2000–16 cannot be represented.

There was a significant log-linear association between national GS and medal tally that persisted after adjustment for sex, age, height, dietary caloric intake, and GDP. The relationship between national GS and log-medal tally is displayed in Fig 1.

Respective Pearson correlation coefficients were 0.67 (p = 0.003), and 0.74 (p = 0.002) for unadjusted GS, and for GS adjusted for age, sex, height, daily dietary caloric intake and national GDP. There was also a positive relationship between country GS, and the natural logarithm of the gold medal tally. The respective Pearson correlation coefficients were 0.57 (p = 0.035), and 0.65 (p = 0.019) for unadjusted GS, and for GS adjusted for age, sex, height, daily dietary caloric intake and national GDP.

There was a modest relationship between national GDP per capita and medal tally. Every USD $1 million increase in per capita GDP is associated with a 0.0031% increase in medal tally (95% CI 0.0012%- to 0.0051%, p = 0.001) and with a Pearson correlation coefficient 0.47 (p = 0.06). There was a strong association between height and medal tally. The unadjusted Poisson regression coefficient was 0.23 (95% confidence interval, CI, 0.097–0.36, p = 0.001), and the unadjusted Pearson correlation coefficient was 0.79 (p = 0.002). We found no significant association between BMI and medal tally (Poisson regression coefficient 0.039, 95% CI -0.21 to 0.29, p = 0.8; Pearson correlation coefficient 0.25, p = 0.3).

## Discussion

The reasons why countries achieve different levels of success in the Olympic Games has, as we note earlier, been the subject of a number of previous studies. These have, however, concentrated on aspects of geography, climate, culture, economic development and politics [1] [2–5]. In parallel, a body of research has been undertaken looking at ethnic and genetic factors favouring certain groups. Thus, following the observation that athletes from some regions of the world excel in particular sports, such as those from East Africa in distance running, several studies have focussed on genetic differences[15]. A study of the relationship between RNA expression and VO_2max_ suggested that 23% of the variance in VO_2max_ could be explained by single-nucleotide polymorphisms[16]. However, for the first time, to our knowledge, we seek to bring these two strands of literature together, looking at how physiological characteristics might influence the overall performance of countries. We believe that, by doing so, we make a discrete, but intriguing contribution to the literature.

It is beyond the scope of our paper to disentangle what is, inevitably, a very complex causal pathway from physiology to national medal scores, for several reasons. First, it is not GS alone that wins medals. Instead, it acts as a proxy for a range of other physiological characteristics that are more directly related to Olympic achievement. Importantly, which characteristics are important will vary among sports, exemplified by the way that different countries excel on race tracks at either sprints (e.g. Caribbean islands) or endurance running (East Africa) [17]. Both involve muscle strength and cardiovascular performance, but in different ways. Second, there are other factors that are important in some sports, such as archery and shooting. We do not know whether grip strength or its correlates affect performance in these sports. Third, it is clear that governments can play a role, through their policies in identifying and nurturing talent, as was the case with Canada and Australia previously [18], and exemplified by the remarkable improvement in performance by, first, China, and then the United Kingdom more recently. Fourth, many wealthy countries benefit from immigration from elsewhere. Finally, there may be country-specific factors, such as the changing cultural and religious acceptability of female participation in sport. We consider some of these issues in more detail below.

To fully understand this complex situation would require a programme of research, employing a range of methods drawing on a variety of disciplines. Moreover, at present, many factors are either unknown or unmeasurable. Thus, we are unaware of any comparative data on grip strength covering countries at such different levels of development prior to the PURE study. Moreover, some associations may vary with context and further quantitative research should look at particular groups of sports, requiring more data points and a longer time period than is currently available.

Turning to the results, we found a surprisingly strong association between mean national GS, and medal tally over the previous four Summer Olympic Games. This association persisted after adjustment for age, sex, height, and country GDP. Consequently, our analysis raises a fascinating hypothesis that a large part of a nation’s success at the Summer Olympic Games is determined by the strength of its entire population, and that the representative athlete is a reflection of this population characteristic. This is something that, we contend, merits further study.

In no way should this analysis detract from the feats of individual athletes in winning medals; their efforts, dedication, and talents are remarkable. These athletes are, nonetheless, born into a socioeconomic and cultural context, with its associated dietary and environmental exposures. Prior to embarking on the training and diet specific to their calling as athletes, they would have been influenced by the food and physical activity environments, as well as healthcare, of their society. Hence, we speculate that favourable environments promote higher population muscle strength, and the elite athlete lies at the extreme end of a Gaussian distribution of strength that is centred upon a higher mean value than countries whose circumstances do not promote strength to the same extent. While this study has found that a 1kg increase in GS might be associated with a 30–40% increase in expected Summer Olympic medal tally, population approaches are unlikely to be implemented with the explicit goal of improving national sporting performance.

There is, however, a compelling public health rationale to try to increase population muscle strength. We have reported that low GS is robustly associated with an increased risk of death, cardiovascular disease, and vulnerability to death in the context of developing a major illness[19]. The present study highlights a potential fringe benefit of improved population health.

We cannot exclude the possibility that there are genetically-based differences between countries with respect to muscle strength. As with any phenotype, a proportion must be heritable. Thus, while muscle strength seems in part genetically determined[20], further research is needed to ascertain the extent to which a common characteristic explains these variations in GS among countries.

There is ongoing debate as to the extent to which athletic performance is modifiable in individuals, and what the asymptotic (best possible) performance in a given sport might be[21, 22]. One systematic review of randomised controlled trials of training interventions for swimming found little evidence that performance could be substantially altered by training techniques[23]. Another study of elite sprinters found that their athletic abilities were remarkable even prior to undertaking rigorous training[24]. The present analysis is consistent with this somewhat fatalistic conception of individual athletic prowess, in that a substantial proportion of the variance in medal tally, as an outcome measure, was accounted for by national muscle strength—a factor that is clearly outside the control of the individual athlete. However, an intriguing question remains, “Can we change muscle strength in countries if we understood its determinants?” We note, for example that the GDP of South Korea began rising appreciably in the late 1970s, and that their Summer Olympic medal tally rose from 6 in Montreal, 1976, to 19 in Los Angeles, and has remained approximately 30 at all Summer Games since.

One obvious way is to change the population through migration. In the London Olympics the United Kingdom benefited not only from a home nation advantage but also from previous decisions to admit Mo Farah from Somalia and Jessica Ennis’s father from Jamaica. Yet with no sense of irony, some of those politicians who lauded their success were, simultaneously, calling for ever greater barriers to immigration. However, while it did not apply in either of these cases, it does raise the complex ethical issue of offering citizenship on the basis of sporting prowess, as happened with unfortunate consequences when South African born Zola Budd was granted British citizenship to run in the 1984 Olympics. Another is to change the composition of the Olympics. The inclusion of baseball in the Olympics between 1992 and 2008 ensured that the USA and Cuba could be sure of winning a medal. Cricket only featured in the Olympics once, in 1900, when the British and French competitors only realised that they were part of the games after the match finished. Had it remained an Olympic sport it seems certain that India’s strikingly low medal total would be higher.

Beyond the limitations noted above, the present analysis was constrained by the limited number of countries studied (it is possible that our results cannot be generalized to all countries), and the lack of data on national sporting policies and those aspects of health and education spending that might influence athletic performance. This study is unable to determine the extent to which population muscle strength is influenced by environmental, as opposed to genetic factors. It is possible that that the relationship between muscle strength and Olympic medal tallies that we observed might be accounted for by unmeasured potential confounders. It is also possible that our findings may not apply to countries that were not studied, among which other factors might be important determinants of Olympic medal success. Lastly, as noted above, muscle strength (in an individual athlete and by extension of a country that he or she represents) may not be the most important determinant of Olympic medal success in many sports where other physical characteristics are better correlated with performance.

The major strength of this study is its novelty. No other single study has measured individuals’ anthropometrics and muscle strength in large numbers across such a wide range of countries. The PURE study therefore offers a unique opportunity to explore the relationship between population characteristics and national sporting accomplishments.

In conclusion, much of the existing literature on success at Olympic Games has focused on the population size, politics, and economy of participating countries. This paper suggests that these earlier researchers may have paid insufficient attention to the physical characteristics of the populations from which the elite athletes are drawn. This gives added meaning to the words of Olympic gold medallist, Scott Hamilton, who is credited with the quotation, “…the Olympic Games is something that belongs to everybody.”

## Supporting Information

S1 Appendix The complete authorship group of the PURE Study. (DOC)

S2 Appendix The Ethics Committees that approved the PURE Study. (DOCX)
