# Supplementary material for: Population Muscle Strength Predicts Olympic Medal Tallies: Evidence from 20 Countries in the PURE Prospective Cohort Study
Source: PLoS One. 2017 Jan 20;12(1):e0169821. doi: 10.1371/journal.pone.0169821 (PMC5249146; doi:10.1371/journal.pone.0169821)
Supplement: S1 Appendix — (DOC) [file pone.0169821.s001.doc]

**S1. PURE Project Office Staff, National Coordinators, Investigators, and Key Staff:**

**Project office (Population Health Research Institute, Hamilton Health Sciences and McMaster University, Hamilton, Canada):** S Yusuf* (Principal Investigator).

S Rangarajan (Project Manager); K K Teo, C K Chow, M O’Donnell, A Mente, D Leong, A Smyth, P Joseph, S Islam (Statistician), M Zhang (Statistician), W Hu (Statistician), C Ramasundarahettige (Statistician), G Wong (Statistician), L Dayal, A Casanova, M Dehghan (Nutritionist), G Lewis, A Aliberti, A Reyes, A Zaki, B Lewis, B Zhang, D Agapay, D Hari, E Milazzo, E Ramezani, F Hussain, F Shifaly, I Kay, J Rimac, J Swallow, L Heldman, M(a) Mushtaha, M(o) Mushtaha, M Trottier, N Aoucheva, N Kandy, P Mackie, R Solano, S Chin, S Ramacham, S Shahrook, S Trottier, T Tongana, W ElSheikh, J Lindeman.

**Core Laboratories**: M McQueen, K Hall, J Keys (Hamilton), X Wang (Beijing, China), J Keneth, A Devanath (Bangalore, India).

**Argentina:** R Diaz*; A Orlandini, B Linetsky, S Toscanelli, G Casaccia, JM Maini Cuneo; **Bangladesh:** O Rahman*, R Yusuf, AK Azad, KA Rabbani, HM Cherry, A Mannan, I Hassan, AT Talukdar, RB Tooheen, MU Khan, M Sintaha, T Choudhury, R Haque, S Parvin; **Brazil:** A Avezum*, GB Oliveira, CS Marcilio, AC Mattos; **Canada:**  K Teo***,** S Yusuf***,** J Dejesus, D Agapay, T Tongana, R Solano, I Kay, S Trottier, J Rimac, W Elsheikh, L Heldman, E Ramezani, G Dagenais, P Poirier, G Turbide, D Auger, A LeBlanc De Bluts, MC Proulx, M Cayer, N Bonneville, S Lear, D Gasevic, E Corber, V de Jong, I Vukmirovich, A Wielgosz, G Fodor, A Pipe, A Shane; **CHILE:** F Lanas*, P Seron, S Martinez, A Valdebenito, M Oliveros; **CHINA:** Li Wei*, Liu Lisheng*, Chen Chunming, Wang Xingyu, Zhao Wenhua, Zhang Hongye, JiaXuan, Hu Bo, Sun Yi, Bo Jian, Zhao Xiuwen, Chang Xiaohong, Chen Tao, Chen Hui, Chang Xiaohong, Deng Qing, Cheng Xiaoru, Deng Qing, He Xinye, Hu Bo, JiaXuan, Li Jian, Li Juan,Liu Xu, Ren Bing, Sun Yi, Wang Wei, Wang Yang, Yang Jun, Zhai Yi, Zhang Hongye, Zhao Xiuwen,Zhu Manlu, Lu Fanghong, Wu Jianfang, Li Yindong, Hou Yan, Zhang Liangqing, Guo Baoxia, Liao Xiaoyang, Zhang Shiying, BianRongwen, TianXiuzhen, Li Dong, Chen Di, Wu Jianguo, Xiao Yize, Liu Tianlu, Zhang Peng, Dong Changlin, Li Ning, Ma Xiaolan, Yang Yuqing, Lei Rensheng, Fu Minfan, He Jing, Liu Yu, Xing Xiaojie, Zhou Qiang, ; **Colombia:** P Lopez-Jaramillo*, PA Camacho Lopez, R Garcia, LJA Jurado, D Gómez-Arbeláez, JF Arguello, R Dueñas, S Silva, LP Pradilla, F Ramirez, DI Molina, C Cure-Cure, M Perez, E Hernandez, E Arcos, S Fernandez, C Narvaez, J Paez, A Sotomayor, H Garcia, G Sanchez, T David, A Rico; **India:** P Mony *, M Vaz*, A V Bharathi, S Swaminathan, K Shankar AV Kurpad, KG Jayachitra, N Kumar, HAL Hospital, V Mohan, M Deepa, K Parthiban, M Anitha, S Hemavathy, T Rahulashankiruthiyayan, D Anitha, K Sridevi, R Gupta, RB Panwar, I Mohan, P Rastogi, S Rastogi, R Bhargava, R Kumar, J S Thakur, B Patro, PVM Lakshmi, R Mahajan, P Chaudary, V Raman Kutty, K Vijayakumar, K Ajayan, G Rajasree, AR Renjini, A Deepu, B Sandhya, S Asha, HS Soumya; **Iran:** R Kelishadi*, A Bahonar, N Mohammadifard, H Heidari; **Malaysia:** K Yusoff*, TST Ismail, KK Ng, A Devi, NM Nasir, MM Yasin, M Miskan, EA Rahman, MKM Arsad, F Ariffin, SA Razak, FA Majid, NA Bakar, MY Yacob, N Zainon, R Salleh, MKA Ramli, NA Halim, SR Norlizan, NM Ghazali, MN Arshad, R Razali, S Ali, HR Othman, CWJCW Hafar, A Pit, N Danuri, F Basir, SNA Zahari, H Abdullah, MA Arippin, NA Zakaria, I Noorhassim, MJ Hasni, MT Azmi, MI Zaleha, KY Hazdi, AR Rizam, W Sazman, A Azman; **OCCUPIED PALESTINIAN TERRITORY:** R Khatib*, U Khammash, A Khatib, R Giacaman; **PAKISTAN:** R Iqbal*, A Afridi, R Khawaja, A Raza, K Kazmi; **PHILIPPINES:** A Dans*, HU Co, JT Sanchez, L Pudol, C Zamora-Pudol, LAM Palileo-Villanueva, MR Aquino, C Abaquin, SL Pudol, ML Cabral; **Poland:** W Zatonski*, A Szuba, K Zatonska, R Ilow**^#^**, M Ferus, B Regulska-Ilow, D Różańska, M Wolyniec; **SAUDI ARABIA:** KF AlHabib*, A Hersi, T Kashour, H Alfaleh, M Alshamiri, HB Altaradi, O Alnobani, A Bafart, N Alkamel, M Ali, M Abdulrahman, R Nouri; **South Africa:** A Kruger*, H H Voster, A E Schutte, E Wentzel-Viljoen, FC Eloff, H de Ridder, H Moss, J Potgieter, AA Roux, M Watson, G de Wet, A Olckers, JC Jerling, M Pieters, T Hoekstra, T Puoane, E Igumbor, L Tsolekile, D Sanders, P Naidoo, N Steyn, N Peer, B Mayosi, B Rayner, V Lambert, N Levitt, T Kolbe-Alexander, L Ntyintyane, G Hughes, R Swart, J Fourie, M Muzigaba, S Xapa, N Gobile , K Ndayi, B Jwili, K Ndibaza, B Egbujie; **Sweden:** A Rosengren*, K Bengtsson Boström, U Lindblad, P Langkilde, A Gustavsson, M Andreasson, M Snällman, L Wirdemann, K Pettersson, E Moberg ; **TANZANIA:** K Yeates*, J Sleeth, K Kilonzo**; TURKEY:** A Oguz*, AAK Akalin, KBT Calik, N Imeryuz, A Temizhan, E Alphan, E Gunes, H Sur, K Karsidag, S Gulec, Y Altuntas; **UNITED ARAB EMIRATES:** AM Yusufali*, W Almahmeed, H Swidan, EA Darwish, ARA Hashemi, N Al-Khaja, JM Muscat-Baron, SH Ahmed, TM Mamdouh, WM Darwish, MHS Abdelmotagali, SA Omer Awed, GA Movahedi, F Hussain, H Al Shaibani, RIM Gharabou, DF Youssef, AZS Nawati, ZAR Abu Salah, RFE Abdalla, SM Al Shuwaihi, MA Al Omairi, OD Cadigal; R.S. Alejandrino; **Zimbabwe:** J Chifamba*, L Gwaunza, G Terera, C Mahachi, P Murambiwa, T Machiweni, R Mapanga.

*National Coordinator

^#^ Deceased
