# Supplementary material for: Population Muscle Strength Predicts Olympic Medal Tallies: Evidence from 20 Countries in the PURE Prospective Cohort Study
Source: PLoS One. 2017 Jan 20;12(1):e0169821. doi: 10.1371/journal.pone.0169821 (PMC5249146; doi:10.1371/journal.pone.0169821)
Supplement: S2 Appendix — (DOCX) [file pone.0169821.s002.docx]

**S2. Ethics Committees that have granted national or regional approval for the conduct of the PURE study**

| **Country** | **Name of ethics review board and institution** |
| --- | --- |
| Argentina | Comité de Etica en Investigación Clínica (CEIC) |
| Brazil | Comissão Nacional de Ética em Pesquisa – Conep approval nº 9047; Comitê de Ética do Instituto Dante Pazzanese de Cardiologia – CEP/Dante approva nº 3310  Institution – Instituto Dante Pazzanese de Cardiologia |
| Chile | Comite de Etica Científica del Servicio de Salud Araucanía Sur |
| Colombia | Comité de Ética en Investigación de la Fundación Cardiovascular de Colombia |
| Canada – Hamilton | Hamilton Integrated Research Ethics Board |
| Canada – Quebec | Comité d’éthique de la recherche de l’Institut Universitaire de Cardiologie et de Pneumologie de Québec |
| Canada – Ottawa | Ottawa Hospital Research Ethics Boards |
| Canada – Vancouver | Office of Research Ethics, Simon Fraser University |
| Sweden | Regional Ethical Review Board of Gothenburg |
| Poland | Institutional review boards at Medical University of Wroclaw |
| Turkey | Marmara University Medical Faculty Ethics Committee-2005  HA Istanbul Göztepe Training and Research Hospital Ethics Committee -2007  Health Authorithy approval-2008 |
| Iran | Ethics Committee, Isfahan Cardiovascular Research Center, Isfahan University of Medical Sciences |
| UAE | Medical Research Committee, Dubai Health Authority. |
| S Africa – Potchefstroom | Faculty of Health Sciences: Ethics sub-committee (Sub-committee of the North-West University Research Ethics Committee) |
| S Africa – Cape Town | Senate Research Committee of the University of the Western Cape, South Africa |
| Zimbabwe | Joint Ethics Research Committee (JREC)  Medical Research Council of Zimbabwe (MRCZ) |
| China | Beijing Hypertension League Institute Ethics Committee |
| Malaysia – UiTM | Research Ethics Committee UiTM, Malaysia |
| Malaysia – UKM | Research Ethics Committee., Universti Kebangsaan Malaysia, Medical Centre, Kuala Lumpur, Malaysia |
| India – Bangalore | St John's Medical College & Hospital Institutional Ethical Review Board |
| India – Jaipur | Institutional Ethics Committee of Monilek Hospital and Research Centre, Jaipur |
| India – Chennai | Institutional Ethics Committee of Madras Diabetes Research Foundation |
| India – Trivandrum | Ethics Committee, Health Action by People |
| India – Chandigarh | Institute Ethics Committee, Post Graduate Institute of Medical Education and Research (PGIMER), Chandigarh |
| Pakistan | Ethical Review Committee. The Aga Khan University |
| Bangladesh | Bangladesh Medical Research Council |
